# Rheological Characterization of Hydrogels from Alginate-Based Nanodispersion

**DOI:** 10.3390/polym11020259

**Published:** 2019-02-03

**Authors:** Francesca Cuomo, Martina Cofelice, Francesco Lopez

**Affiliations:** Department of Agricultural, Environmental and Food Sciences (DiAAA) and Center for Colloid and Surface Science (CSGI), University of Molise, Via De Sanctis, I-86100 Campobasso, Italy; m.cofelice1@studenti.unimol.it (M.C.); lopez@unimol.it (F.L.)

**Keywords:** nanodispersions, alginate, rheology, hydrogels

## Abstract

The interest toward alginate and nanoemulsion-based hydrogels is driven by the wide potential of application. These systems have been noticed in several areas, ranging from pharmaceutical, medical, coating, and food industries. In this investigation, hydrogels prepared through in situ calcium ion release, starting from lemongrass essential oil nanodispersions stabilized in alginate aqueous suspensions in the presence of the nonionic surfactant Tween 80, were evaluated. The hydrogels prepared at different concentrations of oil, alginate, and calcium were characterized through rheological tests. Flow curves demonstrate that the hydrogels share shear thinning behavior. Oscillatory tests showed that the strength of the hydrogel network increases with the crosslinker increase, and decreases at low polymer concentrations. The hydrogels were thixotropic materials with a slow time of structural restoration after breakage. Finally, by analyzing the creep recovery data, the hydrogel responses were all fitted to the Burger model. Overall, it was demonstrated that the presence of essential oil in the proposed hydrogels does not affect the mechanical characteristics of the materials, which are mainly influenced by the concentration of polymer and calcium as a crosslinker.

## 1. Introduction

The study of polymers for functional soft materials has advanced rapidly. Among the various polymers used, alginate is one of the most representative. A great number of applications have been proposed for alginate, ranging from delivery systems, biosensors, soft actuators, and food technology [1,2,3,4,5,6]. In the field of food technology, alginate is used to keep ingredients in foods as a thickener or stabilizer, and to produce gelled and creamy foods, such as ice creams and fruit juices. Alginate is a linear polysaccharide isolated from bacteria and brown algae, and is made of linked residues of (1-4)-linked blocks of poly-β-d-mannuronic acid (M) and poly-α-l-guluronic acid (G). The emulsion/dispersion technology frequently uses an internal gelation process for designing alginate particles [7]. Nanoemulsions or nanodispersions represent a strategy for solubilizing lipophilic molecules in aqueous media and designing new products with enriched functionalities [8]. Usually, the dispersed phase of oil in water nanoemulsions is made of oil droplets, and the addition of a polymer like alginate in the continuous phase gives the systems more stability, due to the thickening agent role of alginate.

The use of alginate is very often associated with the gelation process that occurs in the presence of divalent cations like calcium ions, forming structures known as hydrogels. The term hydrogel refers to a semisolid fluid where a polymer forms a three-dimensional network that swells in water [9,10]. The application fields of such materials are many, and range from clinical practice, biomedical use, food science etc. [11,12]. Many research groups have found interest in the study of alginate hydrogel formation. In 1994, Draget et al. [13] studied the influence of unit sequence and the molecular weight of different alginate molecules on the properties of the relative hydrogels, and they demonstrated that alginates with a high guluronic acid content provided stronger gels compared to alginates rich in mannuronate. Stokke et al. [14] prepared homogeneous Ca-alginate hydrogels through the in situ release of calcium ions, and studied the hydrogels through X-ray scattering and rheology. With this study, it was shown that the final characteristic of the hydrogels are dependent on alginate concentration and composition (ratio M/G), as well as on calcium concentration. Liu et al. [15] studied the rheological parameters of the sol–gel transition of alginate suspensions induced by the in situ calcium release.

Lately, increasing interest has been focused toward complex hydrogels, mainly produced by the gelation of emulsion/nanoemulsion, in order to design more stable materials able to encapsulate and deliver hydrophobic compounds in a hydrophilic network; these have found application in, among others, pharmacological and food fields. In 2010, Josef et al. [16] proposed the use of composite hydrogels prepared through the gelation of the continuous phase of oil-in-water microemulsions for the sustained delivery of hydrophobic drugs. The oil phase of the emulsion allowed the solubilization of hydrophobic drugs, and the crosslinked matrix made the continuous phase, containing alginate, similar to a solid phase. Ketoprofen was used as a model hydrophobic drug. Analyzing the nanostructure by small-angle X-ray scattering (SAXS), the authors demonstrate that the oil droplets exist in the hydrogel matrix, and through release studies they reveal that the rate of drug release is influenced by the network properties.

A successive formulation for the delivery of ketoprofen has been proposed for its transdermal delivery [17]. Different nanoemulsions were selected, and Carbomer 940 was added as the gelation agent for the nanoemulsion. The systems have been characterized for their structure, viscosity, spreadability, and rheological behavior, and permeation studies have demonstrated that the new formulation is more effective for the transdermal delivery of ketoprofen compared with nanoemulsion and marketed formulation.

Very recently, Kaur et al. [18] propose a hydrogel based on nanoemulsion containing Polyphenon 60 and cranberry, developed for the treatment of urinary tract infection and delivered via an intravaginal route. The results of the in vitro studies suggested that the hydrogel could be effective for delivering the active drug toward the organs of the urinary tract.

Moving toward food application for the delivery of therapeutic molecules through the gastrointestinal tract, Nagakawa et al. [19] propose a typology of hydrogels containing oil for encapsulation of curcumin. The hydrogel is prepared with a ternary system made of chitosan, k-carrageenan, and carboxymethylcellulose. Freezing the blend of polymers causes the sol–gel transition with the successful encapsulation of the curcumin. The release behavior of the model food ingredient is investigated in aqueous systems, and the results are influenced by the freezing conditions.

An alginate hydrogel was proposed by Lei et al. [20] to encapsulate nobiletin loaded in nanoemulsion droplets. Nobiletin is a flavonoid isolated from citrus peels with excellent bioactivities, such as anti-inflammatory, anti-cancer, and anti-dementia. Through in vitro release and in vitro digestion studies, the authors conclude that nanoemulsion-filled hydrogels could achieve the sustained release and absorption of nobiletin and prevent its precipitation in the gastrointestinal tract.

Among the lipophilic phases used to formulate nanoemulsions hydrogels, a particular interest is directed to the use of essential oils. Essential oils, besides being able to solubilize lipophilic substances, have antimicrobial and antioxidant properties, characteristics that make them suitable ingredients to preserve cosmetic preparations [21,22]. In 2015, Chen and coworkers [23] demonstrated that the presence of essential oils in a hydrogel formulated for transdermal administration enhances the ibuprofen penetration through skin. The presence of essential oils was demonstrated to be effective against microbial proliferation in composite wound dressing films of sodium alginate containing essential oils [24]. The authors characterize different alginate/glycerol matrices enriched with different essential oils and tested them against *Escherichia coli* and *Candida albicans*. Thanks to their outstanding properties, the use of essential oils is finding room also in the food packaging field, in particular in fresh fruit coating. In a recent application, lemongrass essential oil has been included in alginate-based edible coating formulations [25] applied on fresh-cut apple pieces through gelation, and it has been demonstrated that the presence of the lemongrass essential oil inhibits the proliferation of *E. coli*.

In this scope, understanding of the rheological properties of such gelled systems containing emulsifying or stabilizing agents becomes necessary for improving the systems’ characteristics.

In light of the current increasing interest in composite hydrogels, here we present a study on the rheological characterization of hydrogels prepared from nanodispersions made of an essential oil stabilized in alginate aqueous suspensions by a nonionic surfactant: Tween 80. The rheological characterization of the starting nanodispersions has been recently published [26]. Here, we consider the in situ gelation of the alginate-based nanodispersions and the difference in the mechanical response, along with the variation of oil, alginate, and calcium content.

## 2. Materials and Methods

### 2.1. Materials

Tween 80, d-glucono-δ-lactone (GDL) and ethylenediaminetetraacetic acid (EDTA), and CaCl_2_ were purchased from Sigma Aldrich (St. Louis, MO; USA). Lemongrass (*Cymbopogon nardus*) essential oil (100%) was from Erbamea (Lama di San Giustino PG, Italy), and the food-grade sodium alginate was from Farmalabor (Canosa Di Puglia, Italy).

### 2.2. Preparation of Nanodispersions

Sodium alginate was dissolved in ultrapure water at 70 °C under gentle stirring. Nanodispersions were prepared with a final polyelectrolyte concentration of 0.5 or 1 wt % as the continuous phase, and lemongrass essential oil (EO) at different concentrations (0, 0.1 and 0.5 wt %) as the dispersed phase. All the nanodispersions were stabilized by 1 wt % Tween 80. Coarse emulsions were prepared by mixing the aqueous phase with EO and Tween 80 using a laboratory mixer, T25 digital Ultra-Turrax (IKA, Staufen, Germany), working at 24,000 rpm for four minutes. All the emulsions were then sonicated using an Ultrasonic Homogenizer (Model 300 VT) (BioLogics Inc., Manassas, VA, USA), in order to reduce the dimension of the particles.

### 2.3. Gelation of Nanodispersions

Homogeneous Ca-alginate nanodispersion gels were prepared at room temperature, through the in situ gelation of sodium alginate induced by the release of Ca^2+^ (added as CaCl_2_) from the Ca-EDTA chelate complex. The chelating constant of Ca-EDTA is high at pH 7, where Ca^2+^ is completely complexed, and is extremely low at pH 4, where Ca^2+^ is totally released. The pH was lowered through the slow hydrolysis of GDL. The mole ratio of GDL/Ca-EDTA to adopt in order to yield pH 4 after equilibration for about 24 h after the addition of GDL was calculated through a calibration curve (see Figure S1—Supplementary Materials). The mole ratio selected was GDL/Ca-EDTA = 1.5. Specifically, hydrogels were prepared from 5.2 g of alginate nanodispersions that were mixed with 2.3 mL of Ca-EDTA solution (pH 7) and 2.5 mL of GDL solution, in order to achieve the desired Ca^2+^ after 24 h. CaCl_2_ concentrations were in the range of 4–10 mM. Hydrogels compositions are listed in Table 1, and for the sake of shortness were recalled, considering the calcium levels: low ([Ca^2+^] = 6 mM), medium ([Ca^2+^] = 8 mM), high ([Ca^2+^] = 10 mM), and medium* ([Ca^2+^] = 4 mM).

### 2.4. Rheological Characterization

Rheological measurements of the gelled nanodispersions were carried out through a rotational rheometer, Haake MARS III (Thermo Scientific, Karlsruhe, Germany) 10 days after their preparation. All rheology measurements were made using a 20 mm diameter cone (1° angle) and plate geometry. The temperature was controlled at 20 °C by a cooling and heating system (Phoenix II, Thermo Scientific, Karlsruhe, Germany) in combination with a Peltier heating system. The samples were carefully placed onto the surface of the lower plate, and the upper cone was lowered to a 0.052 mm gap distance. Before testing, samples were left equilibrating for five minutes, in order to allow mechanical and temperature equilibrium. Flow curves were made in control rate mode (CR), varying the shear rate (0.005–500 s^−1^) over 300 sec at 20 °C [27].

For the oscillatory tests, an amplitude strain sweep was carried out at frequency of 1 Hz and deformation ranging from 0.001 to 10. The frequency sweep was made in controlled deformation (fixed from the range of linear viscoelasticity (LVE) determined through amplitude sweep measurements), and in a frequency range from 0.1 to 10 rad/s.

In the oscillatory three-step thixotropy test, step 1 was made in controlled stress oscillation at τ 0.005 Pa at a frequency of 1 Hz for 1 min. Step 2 was made in CR at a shear rate of 1000 s^−1^ for 30 s. Step 3 was made in a controlled stress oscillation at τ 0.005 Pa and a frequency of 1 Hz for 6 min.

Creep recovery experiments were carried out by applying a constant stress (τ = 3 Pa for samples 1–9, and τ = 1 Pa for samples 10–12) for 180 s, following the deformation (loading phase). The stress was then removed, and the recovery phase was registered for 360 s.

## 3. Results and Discussion

**Figure 1:**
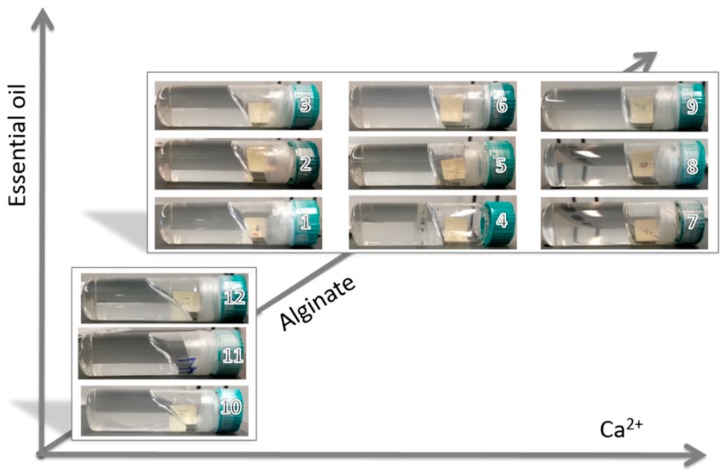
Photographs of alginate-based nanodispersion gels with increasing concentrations of CaCl_2_ (*x*-axis), essential oil (*y*-axis), and alginate (*z*-axis). The CaCl_2_ concentrations ranges from 4 to 10 mM, the essential oil concentration ranges from 0 to 0.5 wt %, and the alginate concentrations were 0.5 or 1 wt %. Tween 80 was kept constant at 1 wt %.

As reported in Table 1, alginate gels based on nanodispersion were prepared by varying CaCl_2_, essential oil, and alginate concentrations. The aspect of the hydrogel formed from the alginate-based nanodispersions is shown in Figure 1, where the vials containing the gels (10 days after the gelification) were placed in horizontal position to display the different gel/air interface profile.

At the top of Figure 1 (from left to right), gels sharing the same content of alginate (1 wt %), but different Ca^2+^ concentration are displayed (samples 1–9). In particular, samples 1, 2, and 3 were gelled with 6 mM of Ca^2+^; samples 4, 5, and 6 with Ca^2+^ 8 mM; and samples 7, 8, and 9 with Ca^2+^ 10 mM. Moreover, samples 1, 4, and 7 did not contain essential oil; samples 2, 5, and 8 contained 0.1 wt % of essential oil; and samples 3, 6, and 9 had an oil content of 0.5%. At the bottom of Figure 1, samples formulated with alginate 0.5 wt %, Ca^2+^ 4 mM, and various oil content (0, 0.1 and 0.5 wt %) are reported (samples 10, 11, and 12, respectively). Remarkably, considering the alginate and Ca^2+^ concentration, these last samples were prepared with the same alginate/Ca^2+^ ratio as samples 4, 5, and 6. As can be seen from Figure 1, all the hydrogels had a very clear aspect, indicating that the essential oil was dispersed in nanometric domains, and according to the sample composition, the gel/air interface was mainly influenced by Ca^2+^ and alginate concentrations. Indeed, high and medium calcium content with a higher alginate percentage gave flat stiff interfaces, while decreasing both the ingredients resulted in hydrogels with less stiff interfaces.

**Figure 2:**
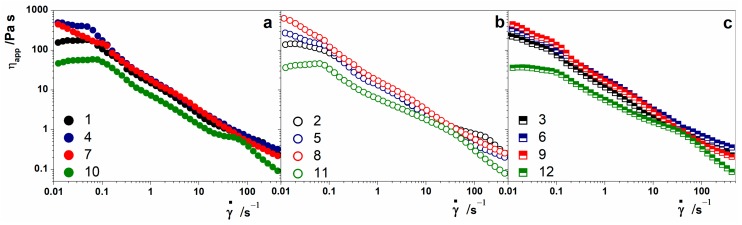
Shear rate dependence of the hydrogels’ shear viscosity. Black symbols indicate samples with 1 wt % of alginate and Ca^2+^ 6 mM, blue symbols indicate samples with 1 wt % of alginate and Ca^2+^ 8 mM, red symbols are for samples with 1 wt % of alginate and Ca^2+^ 10 mM, and green symbols represent samples with 0.5 wt % of alginate and Ca^2+^ 4 mM. (**a**) Hydrogels without essential oil; (**b**) hydrogels with 0.1% essential oil; (**c**) hydrogels with 0.5% essential oil.

Steady-state flow curves of the alginate-based hydrogels demonstrate that all the hydrogels showed shear rate ($\dot{\gamma }$) dependence and shear thinning behavior. From the apparent viscosity curves reported in Figure 2, a Newtonian region corresponding to the steady plateau at low values of $\dot{\gamma }$ is found for some hydrogels. Specifically, hydrogels prepared without oil (Figure 2a) and with low and medium calcium content presented the Newtonian plateau. Samples prepared with less alginate (0.5 wt %) are reported, with green symbols, in Figure 2b,c.

A high Ca^2+^ concentration induced a stronger gelation, which was reflected in the increasing viscosity as the shear rate approached zero, indicating that the fluid does not flow at rest (samples 7, 8, and 9 in Figure 2a–c, respectively). Moreover, the presence of essential oils in hydrogels prepared with low and medium Ca^2+^ concentrations seems to have an effect in the region of low shear rate that causes the loss of the Newtonian plateau [18]. The presence or absence of the Newtonian plateau appears, in some way, to be correlated with the shape of the gel/air interface, i.e., the more the interface is flat, the less the plateau is present.

**Figure 3:**
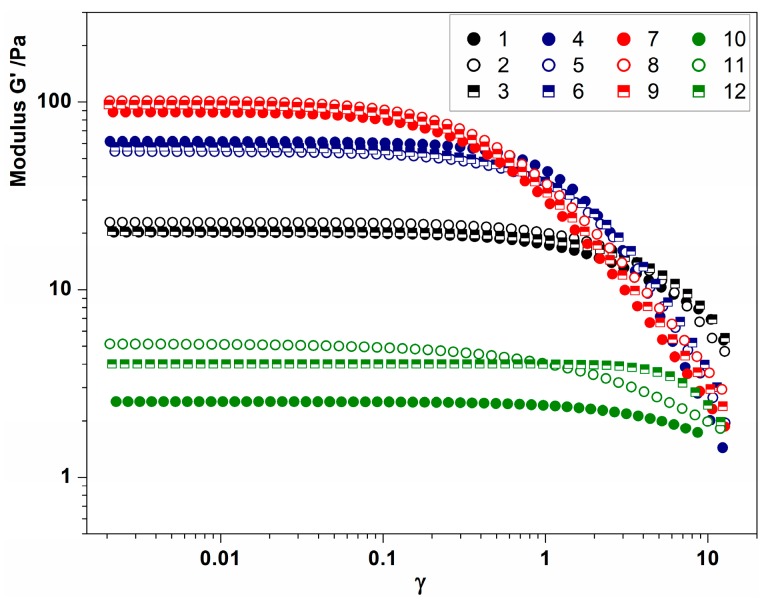
Amplitude strain sweep for hydrogels produced with low Ca^2+^ content (black symbols), medium Ca^2+^ content (blue symbols), high Ca^2+^ content (red symbols) and low alginate, and medium* Ca^2+^ content (green symbols). For detailed sample compositions, see Table 1.

The results of the dynamic oscillation tests gave important information concerning the effect of Ca^2+^ and polymer concentration on the samples’ structures. Strain sweeps confirmed the gel-like behavior, since for all the samples the elastic modulus *G*’ was higher than the viscous modulus *G*” (data not shown). The linear viscoelastic (LVE) region for each hydrogel can be identified, in Figure 3, in the region where *G*’ results are independent of the applied deformation. Considering the hydrogels prepared at 1 wt % alginate, their mechanical strength was evaluated by comparing the values of *G*’ in the LVE region [28], and the results were mainly influenced by the amount of Ca^2+^ used as cross-linker. Hydrogels with low levels of Ca^2+^ had *G*’ values of about 20 Pa, the presence of medium levels of Ca^2+^ increased the value of the elastic modulus around 50 Pa, and high levels of Ca^2+^ provided a further stronger gel, with a *G*’ value around 100 Pa. In all these samples, the strength measured was strongly dependent on the continuous phase, since no difference was evidenced by the presence of the oil-dispersed phase. Very close *G*’ values were also recorded for hydrogels prepared with 0.5% alginate. Although the green symbols seem more differentiated in Figure 3 due to the logarithmic representation, the *G*’ values ranged between 2 and 5 Pa, resulting in those with weaker structures being among the produced hydrogels.

**Figure 4:**
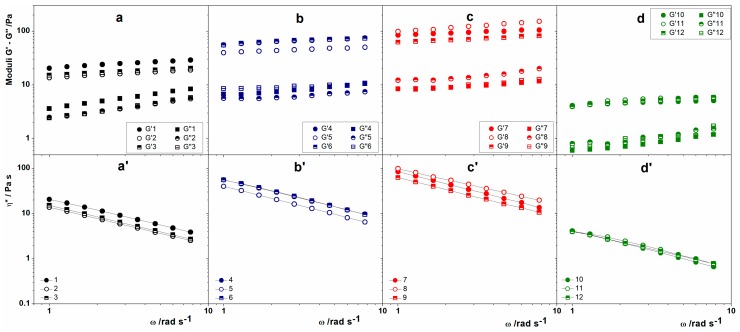
Frequency sweep and complex viscosity (indicated in figure by the letters **a**–**d** and **a’**–**d’**, respectively) for hydrogels produced with (**a**–**a’**) low Ca^2+^ content (black symbols), (**b**–**b’**) medium Ca^2+^ content (blue symbols), (**c**–**c’**) high Ca^2+^ content (red symbols), and (**d**–**d’**) low alginate and medium* Ca^2+^ content (green symbols). For detailed sample compositions, see Table 1.

Frequency sweep tests, performed within the linear viscoelastic region of each hydrogel, enabled the determination of the material frequency dependence, in the range of angular frequency between 0.1 to and 10 rad/sec. The outcomes of the test are reported in Figure 4a–d. The high frequency region corresponds to short-time behavior (simulated by rapid motion); on the other side, the low frequency zone mimic long-term behavior. As can be observed from the figures, all of the hydrogels exhibited a storage modulus (*G*’) that was always higher than the loss modulus (*G*”), denoting that the elastic character is always dominant when a load is applied. A very low dependence of the elastic modulus on the frequency was found for all of the hydrogels, while the *G*” modulus showed a certain dependence in hydrogels having low Ca^2+^ content (Figure 4a) and low alginate content (Figure 4d). This aspect indicates that for these samples with short-term behavior, the viscous response begins to influence the response of the material. In addition, the distance between the moduli did not seem to be influenced by the presence of the oil, representing that the existence of oil dispersed phase does not influence the structure of the hydrogels strongly.

Remarkably, the complex viscosity value η*, reported in Figure 4a’–d’ decreased with the increase of the angular frequency, confirming that all the hydrogels were pseudoplastic fluids with shear thinning behavior.

**Figure 5:**
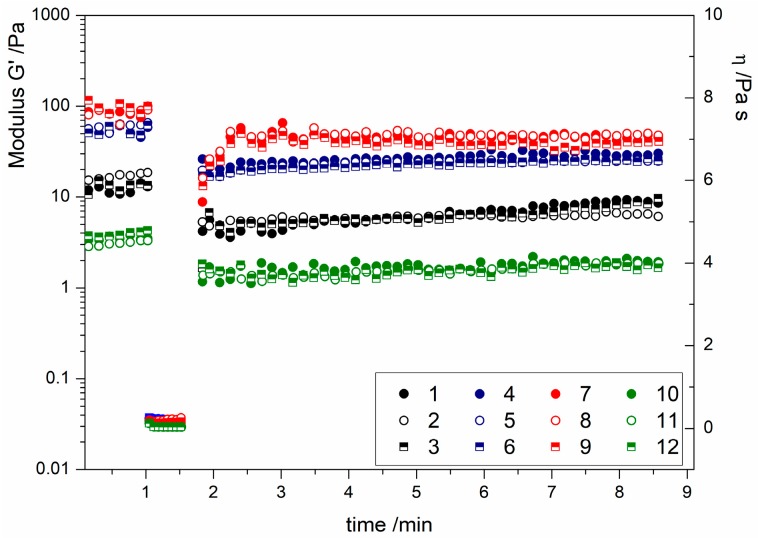
Three-step thixotropy test of hydrogels produced with low Ca^2+^ content (black symbols), medium Ca^2+^ content (blue symbols), high Ca^2+^ content (red symbols), and low alginate and medium* Ca^2+^ content (green symbols). For detailed sample compositions, see Table 1.

In order to understand how the hydrogels’ structures responded to high speed shearing, the thixotropic behavior was studied. The study of thixotropy is characterized by a decrease in the values of rheological parameters, such as the storage modulus *G*’, as consequence of a mechanical load, and the recovery of the initial state upon reduction of the load [29]. The test is made of a first step, where the sample is under very low shear conditions (rest condition), a second phase where the structure is broken under a high shear rate, and a third phase where the conditions of the first phase are applied for a longer time. In the last phase, the structure recovery is observed. The results of the thixotropic tests are illustrated in Figure 5, where the elastic modulus is reported before and after the application of the high shear rate, which caused a dramatic viscosity decrease. As shown, the viscosity values correspond to the symbols collected after the first minute of the experiment.

The obtained results indicate that the structure of all of the hydrogels was broken by the high shear rate application, and no full recovery was observed, indicating that the changes induced by the higher shear step could be permanent.

**Figure 6:**
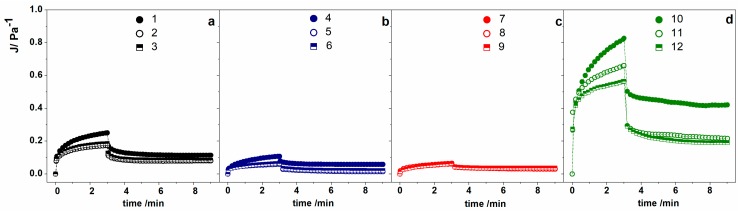
Creep–recovery experiments on hydrogels produced with (**a**) low Ca^2+^ content (black symbols), (**b**) medium Ca^2+^ content (blue symbols), (**c**) high Ca^2+^ content (red symbols), and (**d**) low alginate and medium* Ca^2+^ content (green symbols). For detailed sample compositions, see Table 1.

The viscoelastic properties of the alginate-based hydrogel were further investigated through creep–recovery experiments. Figure 6 illustrates the creep–recovery curves expressed as a compliance variation as a function of time, *J*(*t*). The compliance parameter is given by the ratio of the deformation γ to the applied stress τ:

(1) $$J=\frac{\gamma }{\tau }$$

where the reciprocal of *J* is *G*, which is considered as the rigidity of the material. As a consequence, if a material has a high compliance it has a low rigidity, and vice versa, has high rigidity when exhibiting low compliance. From Figure 6, it is evident that the hydrogels with 0.5 wt % of alginate (Figure 6d) are the less rigid, and moreover that the presence of the oil dispersed phase decreased the compliance by increasing the rigidity. In all the other hydrogels, a higher rigidity is observed according to the increase of Ca^2+^ concentration (Figure 6a–c). The presented data emphasizes that the contribution of the oil phase had a small effect on the rigidity of hydrogels with low and medium Ca^2+^ concentrations, and was negligible in presence of high Ca^2+^ content.

**Scheme 1:**
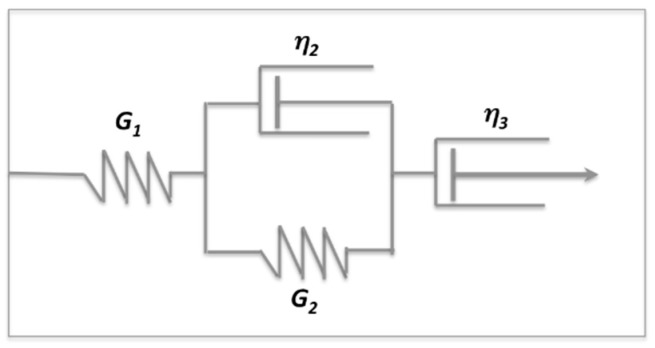
Schematic representation of the Burger model.

In order to analyze quantitatively the materials during the loading step (creep phase), according to the shape of the creep curves the data were fitted to the Burger model (four-parameter Voigt model) [30,31]. The model is made of three elements in a series, as represented in Scheme 1: a spring (elastic element) with a shear modulus *G*_1_; a Kelvin–Voigt element (viscoelastic element), made by a spring and a dashpot in parallel, having shear modulus *G*_2_ and viscosity *η*_2_, respectively; and a dashpot (viscous element) with viscosity *η*_3_.

The Burger model expresses the creep compliance *J*(*t*) with the following equation:

(2) $$J\left(t\right)=\frac{1}{G_{1}}+\frac{1}{G_{2}}\left(1-\exp\left(-\frac{t}{\lambda_{2}}\right)\right)+\frac{t}{\eta_{3}}$$

where *G*_1_ is 1/*J*_1_, which in the creep curve is the time-independent elastic jump; *λ*_2_ is the retardation time of the viscoelastic element; and *η*_3_ is the viscosity of the viscous element. In other words, the compliance, *J*(*t*), in the Burger equation is the sum of an instantaneous elastic response, a viscoelastic (delayed elastic) component, and the unrecovered viscous flow. The elastic response of the viscoelastic element is given by the *G*_2_ parameter from Equation (2) and the viscosity (*η*_2_) is calculated by the retardation time that is given by the ratio of the viscosity to the elastic storage modulus of the second element, *λ*_2_ = *η*_2_/*G*_2_. The parameters obtained by fitting the creep curves to Equation (2) are listed in Table 2.

The elastic component *G*_1_ of the hydrogels from the fittings had a lower value with low alginate concentration, and the *G*_1_ values increased in hydrogels with 1 wt % alginate together with Ca^2+^ content, albeit the values were quite similar for medium and high Ca^2+^ concentrations. The elastic response of the viscoelastic component *G*_2_ follows the same trend as *G*_1_, and a similar trend was also registered for *η*_2_ and for the viscosity of the third element *η*_3_.

The data extrapolated, overall, indicates that the presence of the oil in the composition of the hydrogels at a mechanical level did not cause substantial changes, but the differences observed were principally due to the different calcium levels. In the last column of Table 2, the values of percentage compliance recovery are reported, and were calculated as the ratio between the compliance in the plateau region of the recovery phase and the maximum compliance value at the end of the creep phase. As expected, part of the deformation generated by the loading phase was not recovered because of the viscoelastic character of the hydrogels.

## 4. Conclusions

In this study, the rheological properties of hydrogel-based nanodispersions made of alginate and lemongrass essential oil were analyzed. Different alginate, essential oil, and calcium concentrations were considered. The flow curves of the hydrogels revealed a shear thinning behavior with the presence of a Newtonian plateau at low shear rates in hydrogel formed at low and medium calcium concentrations in the absence of oil, and which persisted in the presence of an oil phase only at low alginate concentrations. Strain sweep tests allowed for determining the LVE region of the hydrogels and the evaluation of the gel strength. Frequency sweep tests confirmed that the characteristics of the hydrogels were mainly influenced by the alginate and calcium concentrations. The thixotropic tests revealed that after the breakage of the hydrogel structure, the complete structure restoration is a slow process; finally, through the creep recovery experiments, the hydrogels were found to be fit with the Burger model.

Among the hydrogel systems studied, the only one that appeared in some way to be influenced by the presence of oil was the one with low alginate concentration, which in creep–recovery experiments deformed differently with the oil concentration increase. In order to give more details on the structural differences, other studies should be carried out.

## Figures and Tables

**Table 1:** Hydrogel compositions. Tween 80 concentration was kept constant at 1 wt %. * These samples were considered with a medium calcium level, because they have the same ratio of alginate/Ca^2+^ as samples 4, 5, and 6.

Hydrogels	Alginate wt %	Essential Oil wt %	CaCl_2_ mM	Calcium Level
1	1	0	6	Low
2	1	0.1	6	
3	1	0.5	6	
4	1	0	8	Medium
5	1	0.1	8	
6	1	0.5	8	
7	1	0	10	High
8	1	0.1	10	
9	1	0.5	10	
10	0.5	0	4	Medium *
11	0.5	0.1	4	
12	0.5	0.5	4	

**Table 2:** Best-fitting parameters calculated for the creep curves and obtained for each hydrogel, according to Equation (2). The last column reports the percentage recovery of deformation.

Hydrogel	*G*_1_ (Pa)	*G*_2_ (Pa)	*η*_2_ (Pa·s)	*λ*_2_ (s)	*η*_3_ (Pa·s)	*R* ^2^	*J_R_* (%)
1	25.62 ± 3.13	7.64 ± 0.28	83.49 ± 7.39	10.92 ± 0.88	2050 ± 91	0.98	45.2
2	52.62 ± 11.29	9.77 ± 0.39	57.25 ± 5.39	5.86 ± 0.50	2889 ± 130	0.98	45.7
3	42.67 ± 8.36	9.55 ± 0.43	57.30 ± 6.02	6.00 ± 0.57	2535 ± 121	0.98	44.9
4	97.93 ± 15.70	22.29 ± 0.87	207.30 ± 19.58	9.30 ± 0.80	2861 ± 96	0.99	54.3
5	132.52 ± 18.33	35.36 ± 1.31	99.36 ± 10.23	2.08 ± 0.20	5013 ± 150	0.98	24.7
6	110.39± 13.91	38.13 ± 1.65	194.46 ± 18.07	5.10 ± 0.41	5205 ± 109	0.98	31.5
7	105.03 ± 5.89	40.29 ± 0.93	576.77 ± 32.88	14.39 ± 0.75	6032 ± 154	0.99	49.3
8	142.84 ± 17.49	36.53 ± 1.14	578.27 ± 27.01	15.83 ± 0.55	4911 ± 100	0.99	48.3
9	107.56 ± 6.46	45.02 ± 1.23	612.27 ± 41.35	13.60 ± 0.84	5151 ± 130	0.98	58.3
10	5.41 ± 0.32	2.73 ± 0.08	35.92 ± 2.74	13.02 ± 0.81	614 ± 19	0.98	48.2
11	9.54 ± 1.21	3.00 ± 0.12	32.70 ± 1.35	10.90 ± 0.11	477 ± 16	0.98	32.6
12	4.48 ± 0.16	4.08 ± 0.13	34.90 ± 2.21	8.56 ± 0.47	1012 ± 66	0.98	33.5

## Supplementary Materials

The following are available online at http://www.mdpi.com/2073-4360/11/2/259/s1, Figure S1: Calibration curve pH vs GDL/Ca-EDTA.

## References

[1] Pawar S.N., Edgar K.J. (2012). Alginate derivatization: A review of chemistry, properties and applications. Biomaterials.

[2] Dang J.M., Leong K.W. (2006). Natural polymers for gene delivery and tissue engineering. Adv. Drug Deliv. Rev..

[3] Tønnesen H.H., Karlsen J. (2002). Alginate in drug delivery systems. Drug Dev. Ind. Pharm..

[4] Cuomo F., Ceglie A., Piludu M., Miguel M.G., Lindman B., Lopez F. (2014). Loading and protection of hydrophilic molecules into liposome-templated polyelectrolyte nanocapsules. Langmuir.

[5] Lee K.Y., Mooney D.J. (2012). Alginate: Properties and biomedical applications. Prog. Polym. Sci..

[6] Cuomo F., Ceglie A., De Leonardis A., Lopez F. (2019). Polymer Capsules for Enzymatic Catalysis in Confined Environments. Catalysts.

[7] Reis C.P., Neufeld R.J., Vilela S., Ribeiro A.J., Veiga F. (2006). Review and current status of emulsion/dispersion technology using an internal gelation process for the design of alginate particles. J. Microencapsul..

[8] Perugini L., Cinelli G., Cofelice M., Ceglie A., Lopez F., Cuomo F. (2018). Effect of the coexistence of sodium caseinate and Tween 20 as stabilizers of food emulsions at acidic pH. Colloids Surf. B Biointerfaces.

[9] Ghica M.V., Hîrjău M., Lupuleasa D., Dinu-Pîrvu C.-E. (2016). Flow and thixotropic parameters for rheological characterization of hydrogels. Molecules.

[10] Ahmed E.M. (2015). Hydrogel: Preparation, characterization, and applications: A review. J. Adv. Res..

[11] Ma S., Yu B., Pei X., Zhou F. (2016). Structural hydrogels. Polymer.

[12] Aarstad O., Heggset E.B., Pedersen I.S., Bjørnøy S.H., Syverud K., Strand B.L. (2017). Mechanical properties of composite hydrogels of alginate and cellulose nanofibrils. Polymers.

[13] Draget K.I., Skjåk Bræk G., Smidsrød O. (1994). Alginic acid gels: The effect of alginate chemical composition and molecular weight. Carbohydr. Polym..

[14] Stokke B.T., Draget K.I., Smidsrød O., Yuguchi Y., Urakawa H., Kajiwara K. (2000). Small-Angle X-ray Scattering and Rheological Characterization of Alginate Gels. 1. Ca−Alginate Gels. Macromolecules.

[15] Liu X., Qian L., Shu T., Tong Z. (2003). Rheology characterization of sol-gel transition in aqueous alginate solutions induced by calcium cations through in situ release. Polymer.

[16] Josef E., Zilberman M., Bianco-Peled H. (2010). Composite alginate hydrogels: An innovative approach for the controlled release of hydrophobic drugs. Acta Biomater..

[17] Arora R., Aggarwal G., Harikumar S.L., Kaur K. (2014). Nanoemulsion based hydrogel for enhanced transdermal delivery of ketoprofen. Adv. Pharm..

[18] Kaur A., Gupta S., Tyagi A., Sharma R.K., Ali J., Gabrani R., Dang S. (2017). Development of Nanoemulsion Based Gel Loaded with Phytoconstituents for the Treatment of Urinary Tract Infection and in Vivo Biodistribution Studies. Adv. Pharm. Bull..

[19] Nakagawa K., Sowasod N., Tanthapanichakoon W., Charinpanitkul T. (2013). Hydrogel based oil encapsulation for controlled release of curcumin by using a ternary system of chitosan, kappa-carrageenan, and carboxymethylcellulose sodium salt. LWT-Food Sci. Technol..

[20] Lei L., Zhang Y., He L., Wu S., Li B., Li Y. (2017). Fabrication of nanoemulsion-filled alginate hydrogel to control the digestion behavior of hydrophobic nobiletin. LWT-Food Sci. Technol..

[21] Halla N., Fernandes I., Heleno S., Costa P., Boucherit-Otmani Z., Boucherit K., Rodrigues A., Ferreira I., Barreiro M. (2018). Cosmetics Preservation: A Review on Present Strategies. Molecules.

[22] Maccioni A., Anchisi C., Sanna A., Sardu C., Dessi S. (2002). Preservative systems containing essential oils in cosmetic products. Int. J. Cosmet. Sci..

[23] Chen J., Jiang Q.-D., Wu Y.-M., Liu P., Yao J.-H., Lu Q., Zhang H., Duan J.-A. (2015). Potential of essential oils as penetration enhancers for transdermal administration of ibuprofen to treat dysmenorrhoea. Molecules.

[24] Liakos I., Rizzello L., Scurr D.J., Pompa P.P., Bayer I.S., Athanassiou A. (2014). All-natural composite wound dressing films of essential oils encapsulated in sodium alginate with antimicrobial properties. Int. J. Pharm..

[25] Salvia-Trujillo L., Rojas-Graü M.A., Soliva-Fortuny R., Martín-Belloso O. (2015). Use of antimicrobial nanoemulsions as edible coatings: Impact on safety and quality attributes of fresh-cut Fuji apples. Postharvest Biol. Technol..

[26] Cofelice M., Lopez F., Cuomo F. (2018). Rheological Properties of Alginate–Essential Oil Nanodispersions Colloids Interfaces. Colloids Interfaces.

[27] Sovrani V., de Jesus L.I., Simas-Tosin F.F., Smiderle F.R., Iacomini M. (2017). Structural characterization and rheological properties of a gel-like β-d-glucan from Pholiota nameko. Carbohydr. Polym..

[28] Patel A.R., Dumlu P., Vermeir L., Lewille B., Lesaffer A., Dewettinck K. (2015). Rheological characterization of gel-in-oil-in-gel type structured emulsions. Food Hydrocolloids.

[29] Mezger T.G. (2006). The Rheology Handbook: For Users of Rotational and Oscillatory Rheometers.

[30] Abdurrahmanoglu S., Okay O. (2010). Rheological behavior of polymer–clay nanocomposite hydrogels: Effect of nanoscale interactions. J. Appl. Polym. Sci..

[31] Suriano R., Griffini G., Chiari M., Levi M., Turri S. (2014). Rheological and mechanical behavior of polyacrylamide hydrogels chemically crosslinked with allyl agarose for two-dimensional gel electrophoresis. J. Mech. Behav. Biomed. Mater..

