# Supplementary material for: Rheological Characterization of Hydrogels from Alginate-Based Nanodispersion
Source: Polymers (Basel). 2019 Feb 3;11(2):259. doi: 10.3390/polym11020259 (PMC6419013; doi:10.3390/polym11020259)
Supplement: Supplementary file 1 [file polymers-11-00259-s001.pdf]

Supplementary Materials: Rheological Characterization of Hydrogels from Alginate-Based Nanodispersion

Francesca Cuomo, Martina Cofelice and Francesco Lopez

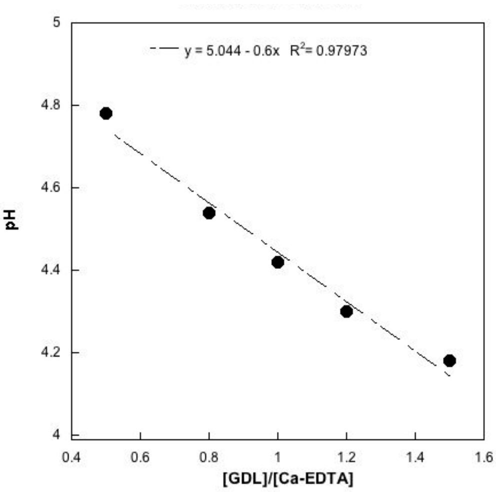

Figure S1. Calibration curve for the pH variation as function of GDL and Ca-EDTA complex.
